# The Role of Dendritic Cell Maturation in the Induction of Insulin-Dependent Diabetes Mellitus

**DOI:** 10.3389/fimmu.2017.00327

**Published:** 2017-03-27

**Authors:** Jacques C. Mbongue, Hector A. Nieves, Timothy W. Torrez, William H. R. Langridge

**Affiliations:** ^1^Center for Health Disparities and Molecular Medicine, School of Medicine, Loma Linda University, Loma Linda, CA, USA; ^2^Ponce Health Sciences University School of Medicine, Ponce, Puerto Rico

**Keywords:** dendritic cells, indoleamine 2,3-dioxygenase, CTB-INS, maturation, type 1 diabetes

## Abstract

Dendritic cells (DCs) are the dominant class of antigen-presenting cells in humans and are largely responsible for the initiation and guidance of innate and adaptive immune responses involved in maintenance of immunological homeostasis. Immature dendritic cells (iDCs) phagocytize pathogens and toxic proteins and in endosomal vesicles degrade them into small fragments for presentation on major histocompatibility complex (MHC) II receptor molecules to naïve cognate T cells (Th0). In addition to their role in stimulation of immunity, DCs are involved in the induction and maintenance of immune tolerance toward self-antigens. During activation, the iDCs become mature. Maturation begins when the DCs cease taking up antigens and begin to migrate from their location in peripheral tissues to adjacent lymph nodes or the spleen where during their continued maturation the DCs present stored antigens on surface MHCII receptor molecules to naive Th0 cells. During antigen presentation, the DCs upregulate the biosynthesis of costimulatory receptor molecules CD86, CD80, CD83, and CD40 on their plasma membrane. These activated DC receptor molecules bind cognate CD28 receptors presented on the Th0 cell membrane, which triggers DC secretion of IL-12 or IL-10 cytokines resulting in T cell differentiation into pro- or anti-inflammatory T cell subsets. Although basic concepts involved in the process of iDC activation and guidance of Th0 cell differentiation have been previously documented, they are poorly defined. In this review, we detail what is known about the process of DC maturation and its role in the induction of insulin-dependent diabetes mellitus autoimmunity.

## Introduction

Insulin-dependent diabetes mellitus (IDDM), also referred to as type 1 diabetes, is a metabolic disease that prevents blood sugar from entering the cells of the body. Onset of IDDM has been identified in children as early as 3–5 years of age. Disease onset results from dysregulated immune cell elimination of the pancreatic islet β-cells the source of insulin production (1, 2). Currently, treatment for IDDM remains palliative based on multiple injections daily of insulin to maintain normal blood sugar levels. The increasing prevalence of IDDM, its progressive complications, and the lack of effective preventive and curative strategies require a greater effort to develop effective and safe methods for persistent restoration of normal blood sugar levels in IDDM patients. So far, no safe and effective treatment is available to control the onset and progression this life-long debilitating disease (3). The loss of β-cell function in IDDM is mediated principally by CD4^+^ and CD8^+^ T cells, and autoreactive effector CD8^+^ T cells (CTLs) were shown to be responsible for islet β-cell destruction in the non-obese diabetic (NOD) mouse and in human IDDM (4–6).

Autoreactive effector thymic (T) cells were shown to transfer IDDM to immune-compromised hosts (4). Based on its wide spread nature and specific autoantigens shown to cause diabetes onset, IDDM became established as a prototypical autoimmune disease. High blood glucose levels are induced in IDDM when dendritic cells (DCs) interact with potential autoreactive effector Th0 cells in peripheral tissues. The DCs were shown to guide differentiation of autoreactive T cells into pro-inflammatory effector cells capable of halting islet insulin biosynthesis by inducing β-cell death (1, 5–12). However, little is known concerning the kinetics and phenotypic changes that occur in DCs during IDDM development in the NOD mouse pancreas. While peri-islet accumulation of conventional myeloid progenitor derived DCs (cDCs) can appear in 4-week-old NOD mice, lymphoid progenitor derived plasmacytoid DCs were shown to surround the islets of Langerhans in mice 10 weeks old (9). The gathering of DCs surrounding the islets occurred along with an influx of lymphocytes (3, 9). Reinforcing the major role for DC participation in diabetes development, the removal of DCs in NOD mice resulted in reduced CD4^+^ T cell activation, stimulation of insulitis, the production of antibodies, and the infiltration of pro-inflammatory Th1/Th17 cells into pancreatic tissues (3, 13). The authors showed that transfer of myeloid DCs (mDCs) into mice without DCs induced IDDM onset. The experimental findings reinforce the importance of DC stimulation of IDDM autoimmunity.

Dendritic cell activation is required for assessment of tolerogenesis. An important feature of tolerogenic DCs is their ability to secrete TGF-β as well as the anti-inflammatory cytokine IL-10 both of which inhibit T lymphocyte secretion of IL-2 and IFN-γ inflammatory cytokines (14–17). During the immunological steady state also referred to as immunological homeostasis, DCs were shown to secrete high levels of IL-10 that modulates activation of neighboring myeloid DCs and promotes the *de novo* induction of tolerogenic DCs. In view of their pivotal role in regulating T cell immunity, DCs may alter the balance between pro-inflammatory T cells and regulatory T cells (Tregs) in IDDM. Studies of mouse IDDM showed that mDCs can possess a hyper-inflammatory phenotype (18). In 1973, Steinman and his colleagues first identified DCs and their ability to stimulate T lymphocytes, which ultimately lead to the realization that DCs were key regulators of both protective immune responses and tolerance to self-antigens (6, 19–21). These experiments demonstrated DC existence in two different states identifiable by morphological, phenotypic, and functional markers and became the first description of DC maturation. With the progress of time, increasing numbers of DC subsets continued to emerge, demonstrating the ability of DCs to differentiate into a variety of specialized antigen-presenting cells (APCs) capable of establishment of immunological tolerance under a variety of tissue conditions.

## Immune Cell-Induced IDDM

Insulin-dependent diabetes mellitus is caused by dysregulated immune cell destruction of the insulin-generating pancreatic islet β-cells. Assault on the β-cells begins with invasion of the islets by mononuclear cells in an acute inflammatory reaction termed “insulitis,” that leads to a progressive destruction of the majority of insulin producing β-cells during disease onset that develops silently over a period of several to many years (8, 22). Clinical symptoms of diabetes generally do not appear until more than 70% of the beta-cell population has been destroyed (22). Apoptosis appears to be the general mechanism by which β-cell death occurs in both rodent IDDM models and in human islets isolated from IDDM patients (22, 23). While the mechanism of β-cell destruction in IDDM remains unclear, it was shown to involve several steps: (1) expression of the TNF type-II transmembrane protein family member Fas ligand on activated CD8^+^ cytotoxic T cells and the Fas receptor present on the β-cell membrane; (2) the release of the cytolytic protein perforin and the proteolytic enzymes granzyme by CD8^+^ T cells; (3) pro-inflammatory cytokine secretion IL-β, TNF-α, and IFN-γ by islet infiltrating T cells; (4) synthesis of reactive oxygen intermediates (ROS) that include nitric oxide secreted by DCs, β-cells, and macrophages; and (5) the activation of immature DCs (22–24). The death of β-cells during insulitis progression is likely triggered by autoantigen-activated DC stimulation of naive autoreactive Th0 cell differentiation into effector T cells that produce a variety of pro-inflammatory cytokines and free radical molecules (23). Additional immune cell types that facilitate IDDM onset include antibody producing B-cells and scavenging macrophages. Autoantibodies are generated by B-cells against early islet autoantigens such as proinsulin and glutamic acid decarboxylase 65, which are the first indicators of β-cell autoimmunity. NOD mice deficient in B cell production due to the presence of Igμ mutations do not develop IDDM (25, 26). In addition, skewing the B cell autoantibody repertoire toward islet antigens, for example, through transgenic expression of insulin-binding immunoglobulin heavy chains in B cells also promoted diabetes development (27).

Despite evidence for the involvement of B cells in IDDM development, their exact functions remain unclear. Autoantibody secretion or antigen presentation to T cells by MHCII receptors has been described as the two most identified functions of B cells (28). These experiments indicate that IDDM is not induced by antibodies or B cells only (28). B cells were shown by Silva et al. to enhance islet autoreactive CD4^+^ T cell promotion of IDDM onset (28). Additional studies have shown that both natural killer cells and macrophages directed to the pancreatic islets by CD4^+^ T cell can also stimulate β-cell death (29).

The primary function of DCs in IDDM is antigen presentation outside and within the islet (13, 30). These studies show that autoantigen presentation is essential for the initiation and continued development of IDDM. Analysis of NOD mouse bone marrow-derived DCs suggests that they synthesize increased levels of IL-12 subunit and NF-κB expression (31, 32).

## DC Activation: Translation from Innate into Adaptive Immunity

Autoimmune disease attack in IDDM resides largely in the progression of DCs from an immature to a mature (active) state. Two distinct mechanisms underlie innate DC activation, both of which result in development of a pro-inflammatory state (33). Distinctions occur in the recognition mechanism for molecules conserved among pathogens. The first mechanism is centered on portions of pathogens recognized by receptors on the surface of DCs commonly referred to as “pathogen-associated molecular patterns” (PAMPs), while the second mechanism involves PAMP-independent DC activation that occurs in DC interactions with self-molecules (autoantigens) or alterations in the cell’s internal composition.

### DC Maturation (Activation)

Dendritic cells are involved in the development of both innate and adaptive arms of the human immune system (34). Immature DCs located in the periphery screen foreign antigens including viruses and microbial pathogens. During uptake and processing of foreign antigens, immature DCs begin to mature and migrate to the spleen or adjacent lymph nodes. During maturation, the DCs begin to synthesize peptides that include major histocompatibility complex (MHC) II molecules, CD40, CD80, and CD86 costimulatory molecules and proteins like CD83 and DC-LAMP that have little known functions (35). Upon maturation, DCs stimulate naïve T cells to differentiate into T-cells capable of generating anti- or pro-inflammatory immune responses (34, 36, 37). Further, DCs in the periphery were shown to induce immune tolerance in communication with the spleen or in the periphery (19, 21). Thus, DCs can be involved in the regulation of a wide variety of immune responses. DCs located in the periphery can differentiate from a state of immaturity residing and capturing passing antigens, to a state of maturity where they become mobile APCs capable of priming naïve T cells. DCs are able to identify and convert antigens into immunogens and express antigen-presenting receptor molecules that include MHCI, MHCII receptors, cytokines, chemokines, costimulatory molecules, and proteases that work in concert to stimulate specific immune responses from naïve T cells. The induction of DC-mediated T cell immune responses may vary from Th2 lymphocytes that induce immunological tolerance to the differentiation of Th1 and Th17 lymphocytes responsible for pro-inflammatory immune responses to pathogens or toxins. Variability in DC-mediated immune responses may depend on myeloid DC1s or lymphoid DC2s and the stage of maturation stimulated by signals from the microenvironment (38, 39). The capacity of DCs to regulate immune responses is directly related to their state of activation. Following antigen processing, environmental factors may stimulate DCs to become mature. These factors may include bacterial-structural or soluble antigens [e.g., lipopolysaccharide (LPS)], inflammatory cytokines, binding to cell surface receptors that include CD40 and members of the TNFR family as well as viral dsRNA. During the development of maturity, DCs go through phenotypic and functional alterations. MHC molecules are moved from endocytic compartments to the surface of the DC during maturation. The uptake of antigens is downregulated, and there is an increase in costimulatory molecules expressed on the cell surface. Further, morphological changes in the DC during maturation include cytoskeleton reorganization, the formation of dendrites, as well as the secretion of proteases, cytokines, and chemokines. During migration from the periphery to the spleen and lymph nodes, the DCs express specific adhesion molecules and chemokine receptors on their cell surface (39).

### PAMP-Dependent DC Activation

During DC maturation, individual DC subsets may perform specialized functions that include changes in migratory patterns, cytokine profiles, kinds of antigens processed, localization in specific tissues, and their presence or absence during the development of inflammation or during immunological homeostasis (3, 19, 21). A function shared by all subsets of DCs is the kind of immune-stimulatory or immune-suppressive signals delivered to cells of the vertebrate immune system that result in the development of immune responses. Two major steps are involved in the development of the described functions. The first step is a requirement for antigen identification. Step 2 involves MHCII antigen presentation accompanied by appropriate secondary signals provided by the DC that stimulate effector T cell activation. It is well known that DCs identify antigens that bind receptors on their cell membrane. Pathogen-specific molecular signals that stimulate a DC response are referred to as PAMPs. If the damaging stimulus comes from molecules in the cell environment, they are referred to as damage-associated molecular patterns (DAMPs). These molecular patterns are composed of molecules common to pathogens or the environment that are not present in the host. The PAMPs provide an external signal to immature DC receptors that initiate a pro-inflammatory response to the infectious pathogen or toxin. The release of uric acid, DNA, or ATP from a cell can be an alert sign to DCs for sensing the presence of cells under stress from microbial invasion or cancer caused necrotic cell death (3, 40). Detection of both DAMPs as well as PAMPs occurs *via* DC surface and internal receptors commonly referred to as pattern recognition receptors (PRRs). These receptors can detect end products of glycation (RAGE), or helicases such as the RIG-I-like receptors (RLRs) are capable of recognizing viral replication in the cell, or NOD-like receptors (NLRs), responsible for intracellular recognition of PAMPs. Finally, toll-like receptors (TLRs) on the surface or internal within the DC are capable of recognizing exogenous and endogenous pathogenic bacterial and viral products.

Viral envelope proteins and ssDNA from the virus genome, the LPS and flagellin proteins from bacteria, zymosan from fungus, and profilin from the parasitic protozoan *Toxoplasma gondii* are typical PAMPs recognized by TLRs on the DC. In contrast, “danger” signals derived from cells or from the environment. For example, the release of molecules such ATP, DNA, or uric acid from a cell can be a warning sign to DCs for sensing the presence of cells under stress from microbial invasion, or cancer caused necrotic cell death and are characteristic DAMPs (3, 40). Several DAMPs such as heat shock and S100 proteins are recognized by RAGE. Other DAMPs, such as ATP and uric acid activate NLRs and induce the formation of inflammasomes that may trigger downstream secretion of pro-inflammatory cytokines such as IL-1β and IL-18. All immunogenic oligonucleotides bind RLRs, which require recognition to induce a signaling response within the DC. The TLRs are the most common of the PRRs. In addition to recognizing a variety of PAMPs and DAMPs, they have been implicated in the initiation of autoimmune diseases. Those TLRs embedded in the DC membrane that include TLR4, TLR5, and TLR1/2, and TLR2/6 heterodimers recognize bacterial membrane components The TLRs 3, 7, 8, and 9 that recognize viral immunogenic oligonucleotides are internal to the DC and are embedded in the endosomal membrane (3, 40). High levels of TLR2 and TLR4 expression on myeloid DCs and TLRs TLR7 and TLR9 present in pDCs may have specific functions in autoimmune disease in which molecular mimicry or autoantibodies to essential nucleic acids are a potential underlying mechanism for autoimmune disease onset.

### PAMP-Independent DC Activation

In 1994, Matzinger proposed an alternative mechanism for DC activation that suggested activation of DCs occurs in response to recognition of host-generated molecules released by cells undergoing the process of necrosis (41). The host endogenous danger signals were considered to mimic PAMPs acting as ligands for PRRs, a concept additionally supported by suspected TLR involvement in mouse skin graft rejection (40, 42, 43). However, because a number of TLRs signal in response to virus infection, a study was carried out to determine whether virus-triggered TLR signaling was required for myeloid DC maturation and induction of antiviral immunity. This study showed that mouse myeloid DCs mature normally after infection *in vivo* or *in vitro* with Sendai virus (SeV) in the absence of TLR3, 7, 8, or 9 signaling (44). The demonstration that DC maturation by SeV requires virus replication in the absence of a TLR response provides evidence for the existence of Matzinger’s intracellular pathway and demonstrates its critical role in DC-mediated antiviral immunity. Elucidation of this pathway establishes a basis for further study into the diversity of mechanisms responsible for virus-induced DC maturation and immunity.

## The Impact of DC Costimulation on the Induction of IDDM

Many studies have shown that antigen delivery to DCs upregulates expression of the DC costimulatory molecules B7-1 (CD80) and B7-2 (CD86) known to induce T cell receptor signaling and promote T cell activation. Synthesis of the glycoprotein DC-LAMP, expressed in the DC lysosomal MHC II compartment, is stimulated following the binding of CD154 to the CD40 receptor (CD40R) found on the surface of DCs. This glycoprotein was suggested to function in promoting antigen processing and transport of antigen-loaded MHC II complexes to the cell surface for presentation to T cells (35). Members of the B7 family are structurally related cell-surface proteins that regulate immune responses by delivering costimulatory or co-inhibitory signals. To date, eight family members have been identified that include the most recognized DC activating costimulatory molecules CD80 (B7-1) and CD86 (B7-2), as well as CD274 [the programmed cell death-1 ligand (PD-L1)], CD273 [the programmed cell death-2 ligand (PD-L2)], CD275 (an inducible costimulatory ligand), CD276 (B7-H3), B7-H4, and B7-H6 (45). Members of the B7 ligands are expressed on lymphoid and other tissues (45). The importance of B7 family signaling in the regulation of immune responses is clear from their demonstrated role in the development of immunodeficiency and autoimmune diseases (45). The diverse signals delivered by B7 ligands emphasize the great potential for treatment of cancer, including leukemia’s and for the treatment of tissue-specific autoimmune diseases like IDDM.

### How Costimulatory Molecules Mediate Inflammation

#### PD-L1 and PD-L2

The PD-L1 and PD-L2 may be found on the surface of immature dendritic cell (iDC), and on mature dendritic cells as well as IFN-γ-treated monocytes and are involved in directing programmed cell death-1 (PD-1), *via* receptor family members CD28 and CTLA4 expressed on the surface of activated T cells (46, 47). Programmed cell death ligand PD-1 contains a tyrosine-linked inhibitory motif. Mice shown to be deficient in PD-1 are susceptible to the development of autoimmune disorders suggesting the existence of a defect in the mechanism of tolerance. Monoclonal Ab blockade of PD-L2 on DCs was shown to enhance T cell proliferation and the production of IFN-γ and IL-10. Blockade of PD-L1 resulted in a more modest form of autoimmunity (36). Inhibition of both PD-L1 and PD-L2 stimulated an additive effect. Monoclonal antibody and Fab-enhanced T cell activation studies showed that both PD-L1 and PD-L2 can inhibit T cell activation (47, 48). The activation of T cells was most enhanced using weak APCs such as iDCs and IL-10-pretreated mDCs, and least activated using strong APCs such as mDCs (46). The experimental findings suggest iDCs contain a balance of stimulatory vs inhibitory molecules that favors inhibition of DC activation, and indicate that PD-L1 and PD-L2 function to reduce the immunostimulatory capacity of iDCs. This result suggests the propensity for an IDDM inflammatory response in DCs may be due to the lack of PD-L1 and/or PD-L2 expression. Both Tregs and the PD-1: PD-ligand (PD-L) pathways are involved in termination of the immune responses (49). Thus, their elimination may result in a loss of immunological tolerance and lead to autoimmunity. The PD-1: PD-L pathway was shown to block the function of self-reactive T cells and protect against autoimmunity in several ways (50). PD-L1 is constitutively expressed by most hematopoietic cells and can be further upregulated upon activation. In contrast, PD-L2 is inducible on DCs, macrophages, and B cells. Both PD-L1 and PD-L2 are ligands of PD-1, which is expressed on activated B cells and T-cells as well as resting T-cells (49, 50).

#### Costimulatory Factors CD86, CD83, CD80, and CD40

The DC surface receptor costimulatory molecules CD86, CD80, CD83, and CD40 generate important signals for stimulating naive T cell differentiation and may inhibit oral tolerance in not only IDDM but also inflammatory bowel disease (IBD) and multiple sclerosis (5, 6, 51, 52).

The DC costimulatory factor CD86 is synthesized and presented on DCs and other APCs that upon T cell recognition of an antigen presented on MHCII, which provides costimulatory signals necessary for T cell activation and survival by acting as a ligand for two receptor proteins on the T cell surface. The CD86 costimulatory factor can bind to either CD28 presented on the T cell (for autoregulation and intercellular association) and to CTLA4 synthesized by the T cell (to attenuate immune suppression and cellular disassociation). Pancreatic islet endothelial cells (ECs) were shown to form a barrier to autoreactive T cell transmigration during the development of islet inflammation in the development of IDDM (53, 54). In a study conducted by Lozanoska-Ochser et al., the goal was to determine the significance of costimulatory molecule synthesis by islet ECs. The authors showed that human islet ECs constitutively express both the ICOS ligand and CD86 (B7-2) but do not constitutively express the costimulatory molecules CD40 or CD80 (B7-1) (55). Examination of the functional activity of islet EC-expressed CD86 was accomplished by coculture of resting islet ECs with CD4 T cells stimulated by ligation with just CD3. Proliferation of T cells in the coculture was blocked by monoclonal antibodies against CD86, demonstrating that costimulatory factor effects are provided to ECs by the expression of CD86 (55, 56). Together, the above data implicate a new function for CD86 on islet ECs and on the vasculature, an essential function for promoting adhesion and expansion of newly activated T cell populations.

The costimulatory protein CD83 was at first shown to be a marker for the maturation of DCs (57). However, further evidence suggests that CD83 may also be involved in the regulation of B cell function, thymic T cell maturation, and peripheral T cell activation (58–60). The expression of CD83 mRNA is differentially regulated in native CD4^+^CD25^+^ regulatory T cells (nTregs). The activation of nTregs was shown to rapidly synthesize large amounts of CD83 (58, 60). These experimental findings show that Treg CD83 expression may be involved in the *in vivo* development of regulatory CD4^+^ T cells.

**Figure 1:**
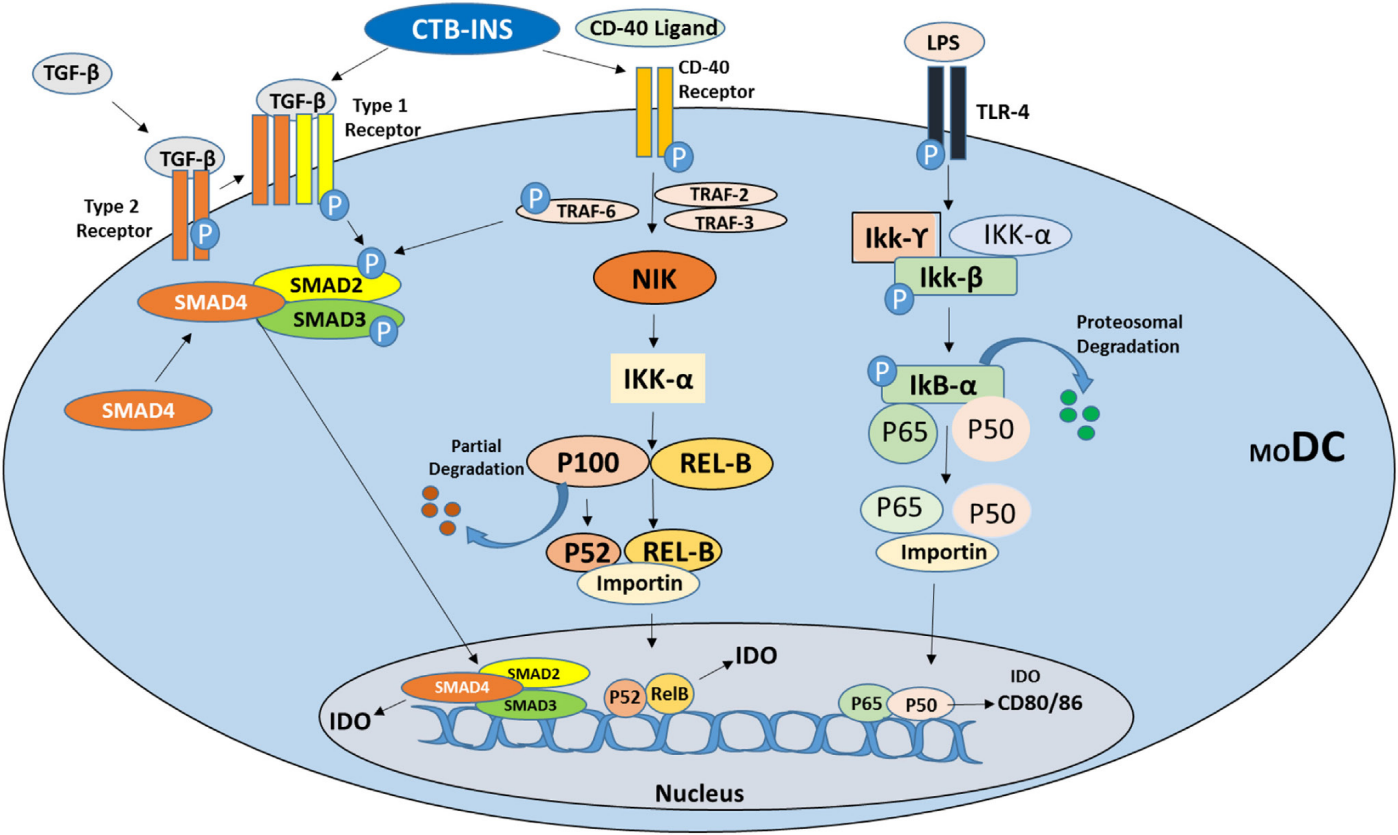
**Suggested signaling pathways for induction of the immunosuppressive catabolic enzyme indoleamine 2,3-dioxygenase (IDO1) in vertebrate dendritic cells (DCs)**. Immunosuppressive cytokine TGF-β and CD40 ligand stimulate tolerogenic DC development by inducing IDO1 synthesis *via* activation of the non-canonical NF-kB signaling pathway as described by Kim et al. (73). Vaccine stimulation of TGF-β may also be involved in the induction of IDO1 biosynthesis through induction of transcriptional activator molecules SMAD2 and 3 phosphorylation ultimately leading to IDO1 biosynthesis. Stimulation of CD40 receptor (TNF-receptor family), by CD40 ligand phosphorylates TRAF2, 3, and 6 proteins stimulating NF-kB non-canonical pathway upregulation of IDO1 biosynthesis directly or through phosphorylation of SMAD2 and 3. Bacterial lipopolysaccharide (LPS) binding to TLR4 initially stimulates IDO upregulation but ultimately triggers DC maturation through upregulation of costimulatory factors CD80/CD86.

A member of the tumor necrosis factor receptor superfamily, costimulatory factor CD40R, can be expressed on the surface of many cell types including DCs, macrophages, B cells, microglia, ECs, epithelial cells, and keratinocytes (61–63). The CD154 ligand for CD40R is briefly synthesized on the membrane of activated CD4^+^ T cells and is also synthesized on immune cells involved in the development of autoimmunity (64, 65). Interactions between CD40R and its ligand CD154 results in movement of CD40R into cholesterol-rich membrane microdomains and the binding of TNFR-associated factors (TRAFs) to its cytoplasmic tail as recently described by Senhaji et al. (66) and others (61, 67–71). The CD40R receptor was also shown to directly bind TRAF2, TRAF3, TRAF5, and TRAF6 and to indirectly associate with TRAF1 (61, 68, 72) (Figure 1). These molecular exchanges result in the upregulation of mitogen and stress-activated protein kinase (MAPK/SAPK) cascades, the activation of transcription factors, the secretion of cytokines, the expansion and morphogenesis of B cells into plasma cells that secrete Ig antibodies, and the development of humoral memory cells. TRAF molecules are associated with overlapping interchangeable and specific CD40-driven interactions (63, 72). For instance, TRAF6 is essential for CD40^−^ activation of JNK and IL-6 synthesis in B cells, while TRAF2 is needed to activate NF-kB, while TRAF3 acts as a negative regulator to inhibit CD40 signaling.

### Suppression of DC Activation

Oral administration of autoantigens to NOD mice was shown to protect against the development of IDDM (74). More recently, the non-toxic B subunit of the cholera enterotoxin (CTB) from *Vibrio cholerae* used as a carrier molecule for linked autoantigens such as proinsulin was shown to induce oral tolerance in NOD mice (75). Studies in our lab and others have shown that oral delivery of CTB conjugated to specific autoantigens greatly enhanced autoantigen protection against the development of autoimmunity in animal models representing several tissue-specific autoimmune diseases (76–78). A C-terminal conjugate of the cholera toxin B subunit (CTB) with proinsulin (CTB-INS) was shown to suppress diabetes onset and partially ameliorate disease progression in NOD mice (79, 80).

The role of CTB in CTB-INS activation of iDCs in regulation of IDDM onset was assessed at an early stage of the human pro-inflammatory immune response (6). In this study, monocyte-derived immature DCs (moDCs) isolated from umbilical cord blood were incubated with CTB-INS. The fusion protein specifically increased plasma membrane expression of DC toll-like receptor 2 (TLR2). Inoculation of immature moDCs with CTB alone stimulated DC costimulatory factors CD86 and CD83 synthesis. In contrast, incubation of moDCs with CTB-INS fusion protein maintained at baseline levels or suppressed biosynthesis of both CD86 and CD83 costimulatory factors below unvaccinated moDC levels. Incubation of monocyte-derived DCs with increasing amounts of insulin stimulated DC maturation by increasing the biosynthesis of both CD86 and CD83 costimulatory factors. Treatment of moDCs with CTB-INS fusion protein inhibited synthesis of the pro-inflammatory cytokine IL-12/23 p40 subunit protein while increasing secretion of the immunosuppressive cytokine IL-10 suggesting that fusion of CTB to insulin may be required for naïve Th0 cell differentiation into immunosuppressive Th2 or Tregs. The data suggest TLR2 may be involved in CTB-INS inhibition of DC-induced IDDM onset. Further, linking CTB to a “self” protein was required for DC stimulation of immune tolerance as co-delivery of CTB, and insulin did not effectively block the biosynthesis of DC costimulatory factors. These findings support the idea that regulation of pancreatic β-cell insulitis depends on both CTB activation of DC TLR2 receptors and processing of CTB linked to the proinsulin autoantigen within the DC.

Disappearance of the monocyte CD14^+^ marker following treatment with the CTB-INS protein suggests the vaccine does not interfere with differentiation of monocytes into immature DCs (81). Further, DC expression of HLA-DR markers remained unaffected (81). The observations that CD86 and CD83 are synthesized in CTB-INS-treated DCs at much lower levels than untreated DCs suggest the vaccine can induce tolerogenesis in CTB-INS-treated DCs (81). The prevention of costimulatory factor CD86 and CD83 biosynthesis in vaccinated DCs was shown to occur simultaneously with biosynthesis of the immunosuppressive tryptophan catabolic enzyme indoleamine 2,3-dioxygenase (IDO1) suggesting their potential linkage in CTB-INS-induced DC tolerance.

Concerning the mechanism of CTB-INS modulation of DC activation, recent experiments indicate that CTB-INS stimulates TNFR signaling and induction of IDO1 biosynthesis in human DCs (73). Inhibition of the TNFR pathway allowed examination of CTB-INS-induced IDO1 biosynthesis in vaccinated DCs. In this recent study, monocyte-derived DCs were incubated with peptides containing amino acid sequences of the TRAF2, 3 and TRAF6 binding sites for CD40. The DCs were then stimulated with CD40 ligand (CD154), and CTB-INS. The addition of CD40-TRAF2, 3 and CD40-TRAF6 blocking peptides inhibited upregulation of IDO1 biosynthesis in response to both CD154 ligand and CTB-INS. The greatest reduction in IDO1 biosynthesis was observed when both TRAF2, 3 and TRAF6 inhibitors were combined (73).

**Figure 2:**
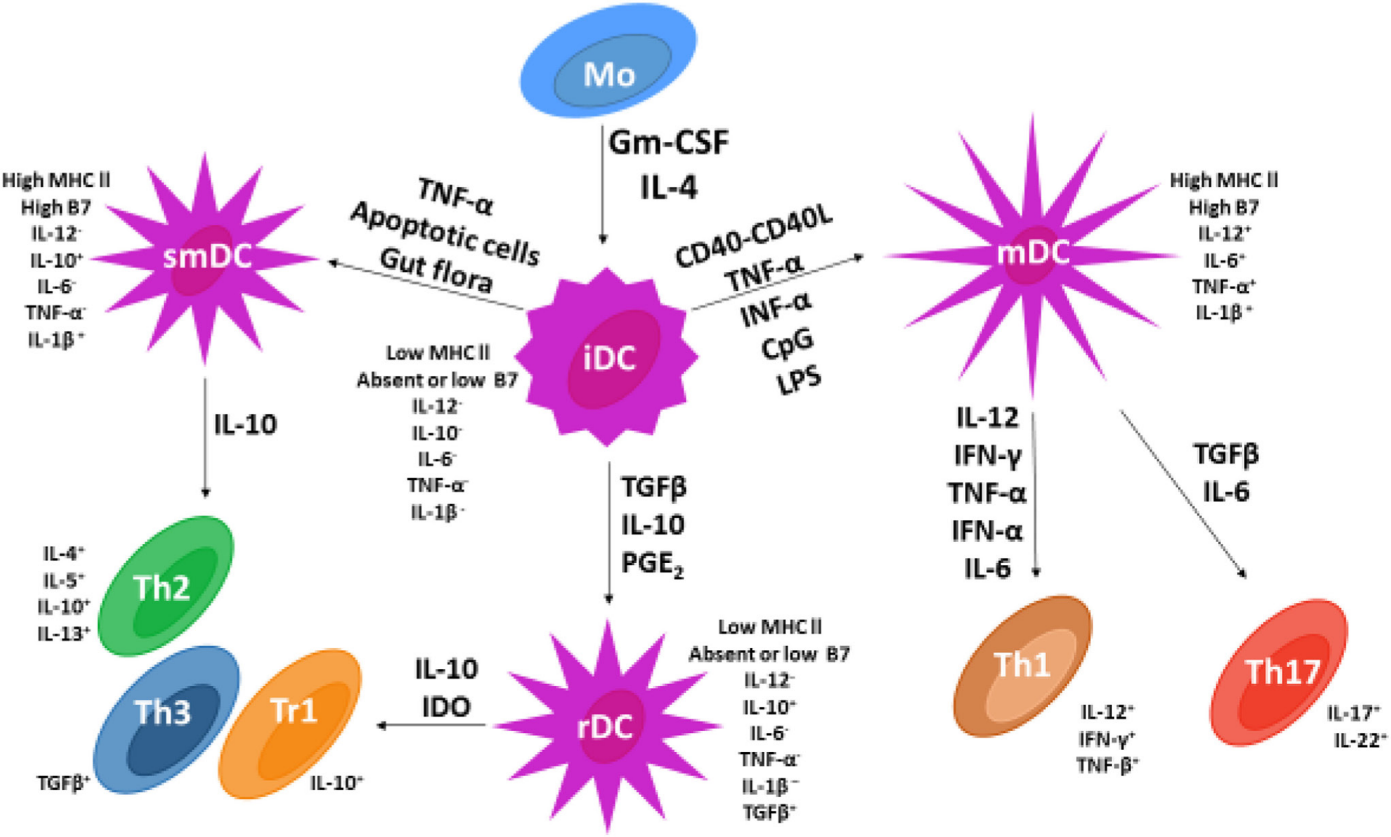
**Dendritic cell (DC) and T cell subsets in vertebrates**. Following the addition of GMCSF and IL-4, monocytes undergo differentiation into immature dendritic cells (iDCs). The addition of various cytokines (adjacent to arrows) permits iDC differentiation into three different DC subsets that include mature dendritic cells, semi-mature dendritic cells, and regulatory dendritic cells (rDCs). Only rDCs and semi-mature dendritic cells express costimulatory factors CD86 and CD80 and secrete the cytokine IL-10 known to stimulate naïve Th0 cell differentiation into Th2 cells and Th3 or Tr1 regulatory T cells. In contrast, mDCs secrete pro-inflammatory cytokines IL-6 and IL-12 that stimulate naïve Th0 cell development into pro-inflammatory Th1 and Th17 lymphocytes.

Based on available data, Figure 2 represents current information on DC maturation and the role DC maturation plays in upregulation of the immune response and development of immune tolerance as a mechanism for inhibition of IDDM autoimmunity.

## Conclusion

The experimental results presented in this review support the development of combinatorial vaccine strategies for the development of safe and effective immune-regulatory therapeutics for prevention and treatment of tissue-specific IDDM autoimmunity. With the progression of time and expanded research efforts, an increased understanding of immunological and molecular mechanisms underlying DC maturation will expand the applications of DCs for understanding the nature of autoimmunity and for construction of a cure for tissue-specific autoimmune disease that for so many generations have plagued mankind.

## Author Contributions

All authors have contributed equally in content in the construction of this review article.

## Conflict of Interest Statement

The authors declare that the research was conducted in the absence of any commercial or financial relationships that could be construed as a potential conflict of interest.
